# Circulating level of fibroblast growth factor 21 is independently associated with the risks of unstable angina pectoris

**DOI:** 10.1042/BSR20181099

**Published:** 2018-09-25

**Authors:** Jing Cheng, Xing Su, Lei Qiao, Chungang Zhai, Wenqiang Chen

**Affiliations:** The Key Laboratory of Cardiovascular Remodeling and Function Research, Chinese Ministry of Education and Chinese Ministry of Health and The State and Shandong Province Joint Key Laboratory of Translational Cardiovascular Medicine, Shandong University Qilu Hospital, Jinan City, Shandong Province, P.R. China

**Keywords:** Coronary artery disease, Fibroblast growth factor 21, Stable angina pectoris, Unstable angina pectoris

## Abstract

There is increasing evidence that serum adipokine levels are associated with higher risks of cardiovascular diseases. As an important adipokine, fibroblast growth factor 21 (FGF21) has been demonstrated to be associated with atherosclerosis and coronary artery disease (CAD). However, circulating level of FGF21 in patients with angina pectoris has not yet been investigated. Circulating FGF21 level was examined in 197 patients with stable angina pectoris (SAP, *n*=66), unstable angina pectoris (UAP, *n*=76), and control subjects (*n*=55) along with clinical variables of cardiovascular risk factors. Serum FGF21 concentrations on admission were significantly increased more in patients with UAP than those with SAP (Ln-FGF21: 5.26 ± 0.87 compared with 4.85 ± 0.77, *P*<0.05) and control subjects (natural logarithm (Ln)-FGF21: 5.26 ± 0.87 compared with 4.54 ± 0.72, *P*<0.01). The correlation analysis revealed that serum FGF21 concentration was positively correlated with the levels of cardiac troponin I (cTnI) (r^2^  =  0.026, *P*=0.027) and creatine kinase-MB (CK-MB) (r^2^  =  0.023, *P*= 0.04). Furthermore, FGF21 level was identified as an independent factor associated with the risks of UAP (odds ratio (OR): 2.781; 95% CI: 1.476–5.239; *P=*0.002), after adjusting for gender, age, and body mass index (BMI). However, there were no correlations between serum FGF21 levels and the presence of SAP (OR: 1.248; 95% CI: 0.703–2.215; *P=*0.448). The present study indicates that FGF21 has a strong correlation and precise predictability for increased risks of UAP, that is independent of traditional risk factors of angina pectoris.

## Background

Coronary artery disease (CAD) comprises acute myocardial infarction (AMI), unstable angina pectoris (UAP), stable angina pectoris (SAP), and sudden cardiac death [1]. The Global Burden of Disease Study reported that CAD affected 110 million people and 8.9 million people died from CAD in 2015 [2]. As the most common cause of death, CAD caused approximately 15.9% of all mortalities globally [3]. CAD has become a global health concern and places a huge burden on the healthcare system. The prognosis for UAP is even worse and associated with higher risk of morbidity and mortality, when compared with SAP. It is really necessary to identify novel biomarkers that are associated with the presence of angina pectoris, especially for UAP.

An increasing amount of data have revealed that adipose tissue can secrete multiple bioactive adipokines [4]. Most adipokines not only play a significant role in metabolic diseases [5], but also exert multiple effects on the function of vessels and heart [6]. Adipokines can accelerate atherosclerosis [7–9] and contribute to the incidence of acute coronary syndrome [10,11]. In recent years, novel adipokines are emerging and their roles in cardiovascular diseases are being investigated. Fibroblast growth factor 21 (FGF21), a member of the endocrine FGFs family, is predominantly produced and secreted by the liver and also expressed in the adipocytes [12]. As an important adipokine, it plays a critical role in adipose growth and differentiation [13]. It is also well studied in physiological and pathological regulation of lipid metabolism and atherosclerotic plaque formation [14]. Circulating FGF21 has been found to be positively associated with carotid intima-media thickness, independent of the well-established risk factors of cardiovascular diseases [15]. Zhang et al. [16] showed that serum FGF21 was independently correlated with the incidence of AMI, and possibly associated with the risk of re-infarction within 1 month after onset. One recent study showed that FGF21 was associated with higher 10-year risk for coronary heart disease (CHD) in patients with no history of diabetes [17]. A prospective cohort study of 1668 CAD patients with a follow-up of approximately 5 years demonstrate that both higher and lower serum FGF21 levels were correlated with higher risks for all-cause mortality, independent of traditional risk factors of cardiovascular diseases [18].

These studies suggest that serum FGF21 level might be a novel biomarker for the diagnosis of CAD. However, circulating levels of FGF21 in patients with angina pectoris have not been well investigated. Thus, the present study aimed to examine the levels of FGF21 amongst patients with SAP, UAP, and control subjects; and further determine the associations between serum FGF21 and the presence of angina pectoris, including both SAP and UAP.

## Methods

### Study population

A total of 197 subjects were recruited between September 2016 and October 2017 at Qilu Hospital, Shandong University in consecutive manner. The patients were categorized into three groups, including: (i) 66 subjects with SAP, diagnosed as paroxysmal exertional chest discomfort that was accompanied with the changes of electrocardiogram in an exercise test; (ii) 76 subjects with UAP, presented as chest pain at rest or aggravated effort type angina within 1 month with the changes of definite ischemic electrocardiogram or recurrent angina pectoris; and (iii) 55 control subjects with normal coronary artery findings and no changes of ECG ischemic ST-T. The exclusion criteria included: (i) patients with congenital heart disease, myocarditis, thromboembolism, cardiomyopathy, collagen disease; (ii) severe kidney and liver diseases; (iii) any malignant diseases; and (iv) some inflammatory diseases, such as septicemia and septicopyemia. All the patients were treated with antiplatelet therapy, β-blockers, statins, and angiotensin-converting enzyme inhibitors. The research has been carried out in accordance with the World Medical Association Declaration of Helsinki. The present study protocol was approved by the Ethics Committee of Qilu Hospital, Shandong University, and informed consent was obtained from each participant.

### Clinical and anthropometric measurements

All the subjects underwent assessment after hospital admission. The general information details about the participants were obtained using a standardized questionnaire via a face-to-face interview with patients, including age, gender, occupation, smoking, alcohol drinking, medication history, disease history, and the presence of other diseases. Smoking status was defined as at least one cigarette per day and lasting for more than 6 months. Alcohol drinking was defined as drinking once or more per week with any type of alcoholic drinks and lasting for at least 6 months. Systolic blood pressure (SBP), diastolic blood pressure (DBP), body weight, and height were measured by trained nurses using a standardized protocol. Body mass index (BMI) was calculated as body weight (kg)/height (m^2^).

### Blood samples measurements

Blood samples were collected after a 12-h fast using separation gel vacuum tube. Serum was obtained by centrifugation at 3000 ***g*** for 15 min in a microcentrifuge at room temperature and stored in aliquots at −80°C until biochemical assays. The biochemical variables, including alanine transaminase (ALT), aspartate aminotransferase (AST), triglyceride (TG), total cholesterol (TC), high-density lipoprotein cholesterol (HDL-c), low-density lipoprotein cholesterol (LDL-c), lipoprotein(a) (Lpa), homocysteine, fasting blood glucose (FBG), blood urea nitrogen (BUN), serum creatinine (SCr), cardiac troponin I (cTnI), creatine kinase-MB (CK-MB), and N-terminal-pro brain natriuretic peptide (NT-proBNP) were tested by standard methods using the Hitachi automatic analyzer 7600-020 (Hitachi) in the routine clinical laboratory. Serum FGF21 concentration was measured by quantitative human ELISA kits (DF2100, R&D Systems, Minneapolis, MN), following the manufacturer’s instructions. The sensitivity of detection was 8.69 pg/ml. The variation coefficients of intra- and inter-assays were <3 and <6%, respectively. All samples were detected in duplicate in a blinded manner.

### Echocardiography

All patients underwent transthoracic echocardiography performed in M-mode and 2D echocardiography by a Philips iE33 ultrasound machine (Philips, America). Left ventricular ejection fraction (LVEF) was calculated from apical four chambers position using the area-length method. Left ventricular end-diastolic diameter (LvEDd) and fractional shortening (FS) were also evaluated by echocardiography in all subjects.

### Statistical analysis

All statistical analyses were calculated with SPSS 21.0 (SPSS, Inc., Chicago, IL, U.S.A.). Results were shown as mean ± S.D., if not otherwise mentioned. All skewed distributions were transformed using natural logarithm (Ln) for analysis. Categorical variables were expressed as frequencies (%) and tested by Chi-square test. Differences amongst continuous variables between two groups were evaluated using two-sample *t* tests, and ANOVA followed by multiple-comparison testing using least significant difference (LSD) *post hoc* analysis was performed amongst three groups. Correlation analyses between serum FGF21 levels and clinical parameters were performed by using Spearman’s correlation analyses. Multivariate logistic regression models were performed to estimate the associations of Ln-FGF21 with the incidence of stable and unstable angina shown as the odds ratios (ORs) and 95% confidence intervals (CIs). We performed receiver operator characteristic (ROC) curve analysis to determine the ability of FGF21 to predict the presence of UAP. *P*-value <0.05 was considered as statistically significant.

## Results

### Clinical characteristics at baseline of the study population

The demographic and biochemical characteristics of the subjects were summarized in Table 1. A total of 197 subjects, including 76 patients with UAP (68.42% males, mean age = 60.05 ± 8.23 years), 66 patients with SAP (54.55% males, mean age = 60.67 ± 9.51 years), and 55 control subjects (38.18% males, mean age = 59.38 ± 9.18 years) were included in the study. There were no differences in history of hypertension, drinking or smoking status, BMI, the actual blood pressure values (including SBP and DBP) amongst the three groups. For the biochemical characteristics, ALT, AST, TC, TG, HDL-c, LDL-c, lipoprotein a (Lp(a)), homocysteine, FBG, BUN, SCr, cTnI, CK-MB, and NT-proBNP were similar amongst the three groups. However, the level of HDL-c was lower in patients with UAP and a higher rate of diabetes was observed in patients with UAP, compared with control subjects. Furthermore, we compared HDL-c and FGF-21 levels between diabetic-UAP and non-diabetic UAP patients. It showed that no significant difference was observed between HDL-c and FGF-21 levels between diabetic and non-diabetic UAP patients (Supplementary Table S1).

**Table 1:** The demographic and biochemical characteristics of the study population

	Controls (*n*=55)	SAP (*n*=66)	UAP (*n*=76)
Age, years	59.38 ± 9.18	60.67 ± 9.51	60.05 ± 8.23
Male, *n* (%)	21 (38.18)	36 (54.55)	52 (68.42)*
BMI (kg/m^2^)	24.66 ± 3.18	24.49 ± 4.44	24.64 ± 3.34
Diabetes, *n* (%)	15 (27.27)	15 (22.73)	28 (36.84)*
Hypertension, *n* (%)	35 (63.64)	29 (43.94)	50 (65.79)
Smoking, *n* (%)	13 (23.64)	28(42.42)	35 (46.05)
Drinking, *n* (%)	14 (25.45)	25 (37.88)	28 (36.84)
SBP (mmHg)	136.05 ± 24.12	139.14 ± 20.69	136.7 ± 20.00
DBP (mmHg)	75.84 ± 13.16	78.95 ± 14.47	79.41 ± 14.33
ALT (U/l)	20.44 ± 19.37	21.55 ± 16.53	26.82 ± 24.47
AST (U/l)	21.38 ± 12.70	20.66 ± 7.74	22.85 ± 15.57
TC (mmol/l)	4.17 ± 1.2	4.37 ± 1.29	9.17 ± 43.13
TG (mmol/l)	1.46 ± 0.84	1.59 ± 1.09	1.54 ± 1.22
HDL-c (mmol/l)	1.18 ± 0.33	1.26 ± 0.33	1.09 ± 0.4*
LDL-c (mmol/l)	2.35 ± 0.86	2.56 ± 0.99	2.23 ± 0.96
Lpa (mmol/l)	16.92 ± 35.55	8.12 ± 20.4	18.63 ± 35.83
Homocysteine (μmoI/l)	13.8 ± 5.87	14.72 ± 5.8	15.05 ± 9.72
FBG (mmol/l)	4.86 ± 1.95	5.05 ± 1.82	5.13 ± 2.73
BUN (mmol/l)	4.67 ± 1.61	4.71 ± 1.54	4.59 ± 1.83
SCr (μmoI/l)	64.19 ± 19.26	66.11 ± 20.00	65.65 ± 23.56
cTnI (ng/l)	6.18 ± 28.1	10.25 ± 46.38	31.56 ± 95.88
CK-MB (ng/ml)	0.67 ± 0.83	0.84 ± 1.05	1.05 ± 1.09

All values are mean ± S.D. *P<0.05 compared with control subjects.

### Correlations between FGF21 levels and clinical variables

**Figure 1:**
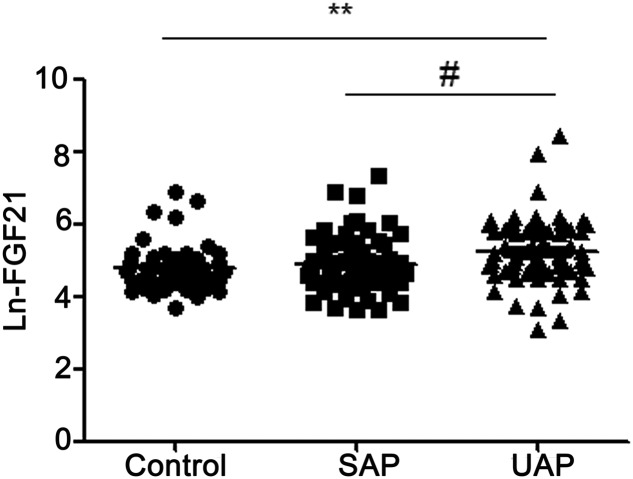
Comparisons of circulating FGF21 level amongst three groups FGF21 was Ln transformed for analysis. **P<0.01 compared with controls; ^#^*P*<0.05 compared with SAP.

**Figure 2:**
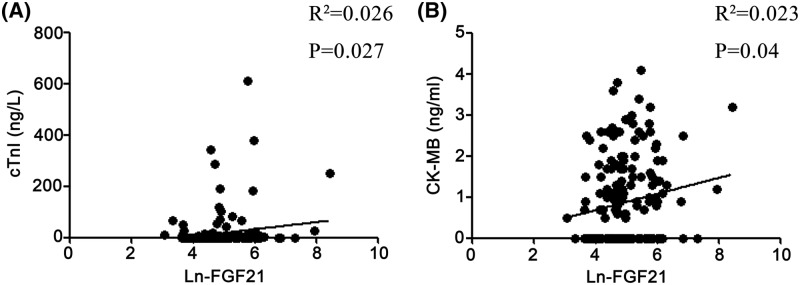
Correlations between FGF21 levels and the concentrations of cTnI and CK-MB in all subjects (**A**) Correlations between FGF21 levels and cTnI, (**B**) correlations between FGF21 levels and CK-MB. FGF21 was Ln transformed for analysis. Correlation analyses were performed by using Spearman’s correlation analyses.

As shown in Figure 1, FGF21 was Ln transformed for analysis, Ln-FGF21 levels on admission were significantly higher in UAP patients than controls (5.26 ± 0.87 compared with 4.54 ± 0.72, *P*<0.01), and also higher than SAP patients (5.26 ± 0.87 compared with 4.85 ± 0.77, *P*<0.05). However, there was no difference in FGF21 levels between SAP patients and control subjects. We further analyzed the correlations between the Ln-FGF21 level and clinical parameters (Table 2). No significant correlations were observed between FGF21 levels and several parameters, including BMI, SBP, DBP, ALT, AST, TG, TC, HDL-c, LDL-c, Lp(a), homocysteine, FBG, BUN, SCr, and NT-proBNP. However, the correlation analysis revealed that serum FGF21 concentration was positively correlated with the levels of cTnI (r^2^  =  0.026, *P*=0.027) and CK-MB (r^2^  =  0.023, *P *=0.04) (Figure 2). Correlation analysis was further performed between FGF21 and clinical variables in UAP patients. It indicated no significant correlations between FGF21 and clinical variables in UAP patients (Supplementary Table S2).

**Table 2:** Correlations between FGF21 levels and clinical variables

Variables	All (*n*=197)	
	r	*P*-value
Age, years	0.01	0.889
BMI (kg/m^2^)	0.02	0.786
SBP (mmHg)	−0.050	0.484
DBP (mmHg)	−0.042	0.555
ALT (U/l)	−0.026	0.727
AST (U/l)	0.103	0.161
TC (mmol/l)	0.022	0.761
TG (mmol/l)	0.019	0.8
HDL-c (mmol/l)	−0.036	0.628
LDL-c (mmol/l)	−0.076	0.298
Lpa (mmol/l)	0.015	0.837
Homocysteine (μmoI/l)	0.017	0.814
FBG (mmol/l)	0.095	0.198
BUN (mmol/l)	0.001	0.996
SCr (μmoI/l)	−0.051	0.493
cTnI (ng/l)	0.163	0.027*
CK-MB (ng/ml)	0.152	0.039*

*P<0.05.

### Serum FGF21 was independently associated with the presence of UAP

**Figure 3:**
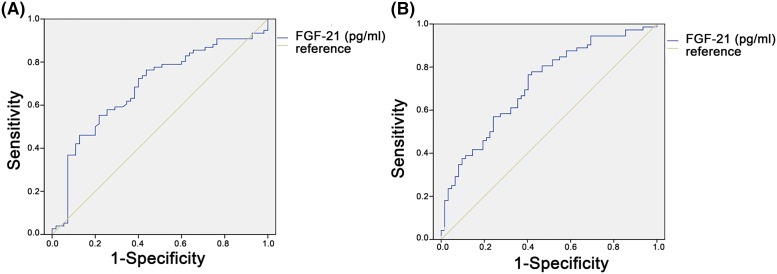
ROC curve of the predictive value of FGF21 for the presence of UAP (**A**) The ROC curve for the prediction of UAP compared with healthy controls by FGF21. The AUC is 0.711 (*P*<0.001); (**B**) ROC curve for the prediction of UAP compared with SAP subjects by FGF-21. The AUC is 0.720 (*P*<0.001).

Next, to determine the independent predictors of the risks of angina pectoris, we performed multiple logistic regression analyses to determine the associations of FGF21 with the incidence of stable and unstable angina (Table 3). FGF21 was independently associated with the presence of angina pectoris (OR: 1.752; 95% CI: 1.081–2.839; *P*=0.023), and UAP (OR: 2.781; 95% CI: 1.476–5.239; *P*=0.002) after adjusting for gender, age, and BMI. After adjusting for diabetes and LDL-c, the association between FGF21 levels and the risks of angina pectoris and UAP was not changed. However, FGF21 was not independently associated with the presence of SAP in a model where variables studied included gender, age, BMI, diabetes, and LDL-c. ROC analysis was performed to determine better cut-off values for FGF-21 with sensitivity, specificity, and area under the curve (AUC) for UAP in comparison with control subjects and SAP patients, respectively. The AUC of ROC curve for the prediction of UAP compared with control subjects by FGF21 is 0.711 (*P*<0.001), and the AUC of ROC curve for the prediction of UAP compared with SAP patients by FGF-21 is 0.720 (*P*<0.001) (Figure 3).

**Table 3:** Multiple logistic regression analysis between FGF21 levels and the presence of angina pectoris

	Ln-FGF21, ORs (95% CI)/*P*-value			
	Model 1	Model 2	Model 3	Model 4
**UAP**	2.781 (1.476–5.239)/*P*=0.002	2.773 (1.472–5.225)/*P*=0.002	2.711 (1.437–5.114)/*P*=0.002	2.766 (1.457–5.253)/*P*=0.002
**SAP**	1.248 (0.703–2.215)/*P=*0.448	1.297 (0.724–2.322)/*P*=0.382	1.323 (0.734–2.385)/*P*=0.352	1.361 (0.726–2.55)/*P*=0.336
**Angina pectoris**	1.752 (1.081–2.839)/*P*=0.023	1.771 (1.089–2.878)/*P*=0.021	1.753 (1.079–2.848)/*P*=0.023	1.865 (1.125–3.089)/*P*=0.016

Model 1: adjusted for age, gender, BMI; Model 2: Model 1 + diabetes; Model 3: Model 2 + smoking history; Model 4: Model 3 + LDL-c.

## Discussion

In our study, we provided evidence that circulating level of FGF21 was significantly increased in patients with UAP, and serum FGF-21 level was positively correlated with the levels of cTnI and CK-MB. Our further analyses indicated that a high level of FGF21 was independently associated with the presence of UAP. Several studies have showed that a high level of circulating FGF21 was found in patients with CAD. However, these studies were mainly focussed on the relationships between FGF21 and the incidence of AMI [16,18-20]. Our present study indicated for the first time that circulating FGF21 may be a novel biomarker for the presence of UAP but not for SAP. This finding is independent of traditional risk factors of angina pectoris.

Epidemiological studies demonstrated that circulating FGF21 levels were positively associated with the onset of multiple cardio-metabolic diseases in humans. Serum FGF21 levels were significantly increased in patients with insulin resistance and T2DM [21]. One recent study found that FGF21 level was an independent predictor of the raised incidence of CHD and may confer higher risks of CHD in T2DM subjects [22]. Elevated level of circulating FGF21 was correlated with the incidence of hypertension in community-dwelling adults [23] and dyslipidemia in CHD patients [24]. Elevated serum FGF21 concentrations were also correlated with hepatic fat content in subjects with non-alcoholic fatty liver disease [25]. Additionally, Kralisch and Fasshauer [26] showed that FGF21 level was increased in obese animals and it was positively associated with BMI in humans. Fisher et al. [27] also found that obese mice had elevated endogenous FGF21 levels and they responded poorly to exogenous FGF21. Consistent with previous studies, we found that circulating FGF21 was positively associated with the presence of UAP. Increasing evidence has proposed that obesity, insulin resistance, and other metabolic diseases are thought to cause a state of FGF21 resistance in both rodents and humans [28,29]. It indicated FGF21 resistance condition may explain the phenomenon of high serum FGF21 levels in pathological conditions of the heart, such as acute coronary syndrome [14]. The term ‘FGF21 resistance’ was first used to describe increased circulating FGF21 levels concomitant to decreased FGF21 receptor complex expression in white adipose tissue of obese mice [27]. Patel et al. [30] also found that increased FGF21 protein expression and serum levels in chronic diet induced obese rats. The underlying mechanism may be caused by disrupted FGF21-FGFR1-β-Klotho signaling and decreased extracellular signal-regulated kinase 1/2 (ERK1/2), protein kinase B (Akt), and AMP-activated protein kinase (AMPK) phosphorylation under the high level of FGF21 [30]. These findings indicate impaired FGF21 signaling cascades in obesity, and that the feedback system allowed the increased production of FGF21 to compensate for the dysfunction of FGF21 receptor signaling. This condition is similar to ‘insulin resistance’, that the impairment of insulin receptor and signaling cascades was found with elevated serum insulin concentration [31,32]. It indicates that FGF21 is increased by a number of stimuli, including oxidative stress, lipopolysaccharide, ethanol, and diets. Accordingly, FGF21 levels can be normalized rapidly, when these stimuli are removed [33]. ‘FGF21 resistance’ was also supported by those patients who had coronary angiography proved that CAD displayed a significantly higher fasting FGF-21, when compared with patients who had no history of admission for coronary angiography [17]. The increase in circulating FGF21 levels might also reflect a protective compensatory response to insulin resistance, hyperlipidemia, and the increase in systemic inflammation in patients with atherosclerotic diseases [34]. Therefore, high serum FGF21 levels in adverse metabolic dysregulation and acute coronary syndrome, such as UAP may be explained by FGF21 resistance conditions. As a novel adipokine, FGF21 plays a critical role in cardio-metabolic diseases, including obesity, diabetes, and cardiovascular diseases. FGF21 is predominantly produced by the liver, but also to a lower extent by adipose tissue, skeletal muscle, and pancreas [12]. It also showed that FGF21 could be synthesized by cardiomyocytes in the heart [35]. To activate FGF21 signaling, FGF21 binds to FGFR1c and FGFR3, and also utilizes b-Klotho as its co-receptor, to form the FGFR/b-Klotho complex [36,37]. Planavila et al. [38] found that FGF21 protects against isoproterenol-induced cardiac hypertrophy by promoting fatty acid oxidation and activating antioxidative pathways. FGF21 also protects the heart from ischemic–reperfusion injury and myocardial infarction by activating Akt-glycogen synthase kinase (GSK)-3β-caspase-3 dependent pathway [39]. Moreover, FGF21 deficiency exacerbated the development of diabetic cardiomyopathy by aggravating cardiac lipid accumulation [40]. In addition, it reported that inflammation plays a critical role of total cardiovascular disease, ischemic heart disease, and myocardial infarction [41,42]. Higher levels of circulating interleukin (IL)-8 were associated with larger infarct size and more adverse clinical outcomes in patients with ST-segment elevation myocardial infarction [43]. Vieira et al. [44] showed targetting of the lymphatic/immune cell axis as a therapeutic paradigm to promote immune modulation and heart repair. One recent study showed that FGF-21 has a potential role in anti-inflammation and immunoregulation, that elevated IL-10 production to correct lipopolysaccharide-induced inflammation [45]. FGF21 deficiency was also found to induce cardiac hypertrophy by promoting oxidative stress, pro-inflammatory pathways, cardiac fibrosis, and cardiac metabolism impairment [35,38]. However, in the present study, a high level of FGF21 was detected in patients with UAP. We speculated that ‘FGF21 resistance’ condition may be existed in patients with UAP, and thus higher FGF21 level may be associated with the presence of UAP by activating several pathways related with oxidative stress and cardiac metabolism. However, the underlying mechanisms need further evaluation.

To our best of knowledge, this is the first study to determine the relationship between serum FGF21 level and the risk of angina pectoris, including both SAP and UAP. It indicates that circulating FGF21 may be deemed as a novel biomarker for the incidence of UAP, but not of SAP. However, the study has several limitations: first, the sample size was relatively small, and we aim to recruit more subjects and enlarge the sample size. Second, it is a cross-sectional study, and hence the casuality between FGF21 level and the incidence of angina pectoris cannot be implied. Third, the long-term prognosis of angina pectoris was not evaluated; thus, a well-controlled longitudinal study is needed to determine the causality between FGF21 level and the development of angina pectoris. In addition, our study was a cross-sectional study that is difficult to determine FGF21 resistance in our study. Our further study will aim to determine FGF21 sensitivity in UAP via insulin tolerance test following co-injection of insulin and FGF21 or weight loss and energy expenditure. Although there are some limitations, our present study is still important as it provides an important basis for further studies about adipokine levels in patients with UAP.

## Conclusion

In conclusion, our present study is novel in showing that circulating FGF21 may be a novel biomarker for the presence of UAP independent of traditional risk factors of angina pectoris. As there is increased risks of morbidity and mortality in angina pectoris, a better understanding of the relationships between FGF21 and the incidence of angina pectoris will be of great benefit. Thus, high-quality, large-scale clinical studies are necessary to confirm the role of FGF-21 in the presence of UAP.

## Supporting information

**Table S1:** FGF21 and lipid levels in diabetic and non-diabetic UAP patients.

**Table S2:** Correlation analysis between Ln-FGF21 and clinical parameters in UAP patients.

## Abbreviations

Akt: protein kinase B
ALT: alanine transaminase
AMI: acute myocardial infarction
AST: aspartate aminotransferase
AUC: area under the curve
BMI: body mass index
BUN: blood urea nitrogen
CAD: coronary artery disease
CHD: coronary heart disease
CK-MB: creatine kinase-MB
cTnI: cardiac troponin I
DBP: diastolic blood pressure
FBG: fasting blood glucose
FGF21: fibroblast growth factor 21
HDL-c: high-density lipoprotein cholesterol
IL: interleukin
LDL-c: low-density lipoprotein cholesterol
Ln: natural logarithm
Lp(a): lipoprotein(a)
NT-proBNP: N-terminal pro brain natriuretic peptide
OR: odds ratio
ROC: receiver operator characteristic
SAP: stable angina pectoris
SBP: systolic blood pressure
SCr: serum creatinine
TG: triglyceride
TC: total cholesterol
UAP: unstable angina pectoris
