# Supplementary material for: Circulating level of fibroblast growth factor 21 is independently associated with the risks of unstable angina pectoris
Source: Biosci Rep. 2018 Sep 25;38(5):BSR20181099. doi: 10.1042/BSR20181099 (PMC6153373; doi:10.1042/BSR20181099)
Supplement: Supplementary file 1 [file bsr20181099_Supp1.pdf]

Table S1. FGF21 and lipid levels in diabetic and non-diabetic UAP patients.

	Diabetic UAP (n=28)	Non-diabetic UAP (n=48)	*p* value
**Ln-FGF21**	5.33±1.00	5.21±0.79	0.606
**TC (mmol/L)**	3.67±1.99	3.85±1.05	0.666
**TG (mmol/L)**	1.26±0.68	1.69±1.42	0.153
**HDL-c (mmol/L)**	0.97±0.53	1.16±0.29	0.096
**LDL-c (mmol/L)**	2.16±1.26	2.26±0.76	0.719

All values are means ± SD; Ln-FGF21: FGF21 was natural-logarithm transformed (Ln) for analysis; TC: total cholesterol; TG, triglycerides; HDL-c: high-density lipoprotein cholesterol; LDL-c: low-density lipoprotein cholesterol; *p* value was by t-test for continuous variables.

Table S2. Correlation analysis between Ln-FGF21 and clinical parameters in UAP patients.

Variables	All (n=76)	
	r	*p*
Age, y	-0.006	0.960
BMI (kg/m2)	0.039	0.737
SBP (mmHg)	0.013	0.908
DBP (mmHg)	0.074	0.526
ALT (U/L)	-0.086	0.468
AST (U/L)	0.171	0.148
TC (mmol/L)	-0.082	0.495
TG (mmol/L)	-0.106	0.347
HDL-C (mmol/L)	0.057	0.629
LDL-C (mmol/L)	-0.120	0.311
Lpa (mmol/L)	0.081	0493
Homocysteine (μmol/L)	0.064	0.590
FPG (mmol/L)	0.066	0.583
BUN (mmol/L)	-0.126	0.293
SCr (μmol/L)	-0.162	0.175
cTnI (ng/L)	0.192	0.106
CK-MB (ng/ml）	0.193	0.104

BMI, body mass index; SBP, systolic blood pressure; DBP, diastolic blood pressure; ALT, alanine aminotransferase; AST, aspartate aminotransferase; TC: total Cholesterol; TG, triglycerides; HDL-c: high-density lipoprotein; LDL-c: low-density lipoprotein cholesterol Lpa: lipoproteins a; FPG: fasting plasma glucose; BUN: blood urea nitrogen; SCr: serum creatinine; cTnI: cardiac troponin I; CK-MB: creatine kinase-MB; Pearson correlation analysis was used.
